# Auricular Acupressure Combined with an Internet-Based Intervention or Alone for Primary Dysmenorrhea: A Control Study

**DOI:** 10.1155/2013/316212

**Published:** 2013-04-03

**Authors:** Mei-Ling Yeh, Yu-Ling Hung, Hsing-Hsia Chen, Jaung-Geng Lin, Yu-Jen Wang

**Affiliations:** ^1^School of Nursing, National Taipei University of Nursing and Health Sciences, Taipei 112, Taiwan; ^2^Taipei Municipal First Girls' Senior High School, Taipei 100, Taiwan; ^3^Department of Applied Mathematics, Chung-Yuan Christian University, Chungli 320, Taiwan; ^4^School of Chinese Medicine-Acupuncture Science, China Medical University, Taichung 404, Taiwan; ^5^Department of Nursing, Chang Gung Universityof Sciences and Technology, Taoyuan 333, Taiwan

## Abstract

*Background*. Primary dysmenorrhea is prevalent in adolescents and young women. Menstrual pain and distress causes poor school performance and physiological damage. Auricular acupressure can be used to treat these symptoms, and Internet-based systems are a flexible way of communicating and delivering the relevant information. *Objective*. This study investigates the effects of auricular acupressure (AA) alone and combined with an interactive Internet-based (II) intervention for the management of menstrual pain and self-care of adolescents with primary dysmenorrhea. *Design*. This study adopts a pretest/posttest control research design with a convenience sample of 107 participants. *Results*. The outcomes were measured using the short-form McGill pain questionnaire (SF-MPQ), visual analogue scale (VAS), menstrual distress questionnaire (MDQ), and adolescent dysmenorrheic self-care scale (ADSCS). Significant differences were found in ADSCS scores between the groups, and in SF-MPQ, VAS, MDQ, and ADSCS scores for each group. *Conclusion*. Auricular acupressure alone and a combination of auricular acupressure and interactive Internet both reduced menstrual pain and distress for primary dysmenorrhea. Auricular acupressure combined with interactive Internet instruction is better than auricular acupuncture alone in improving self-care behaviors.

## 1. Introduction

The rate of primary dysmenorrhea in adolescents and youth worldwide is relatively high: 83.2% in Singapore [1], 82% in Korea [2], 73.3% in Taiwan [3], 72.7% in Turkey [4], 71.1% in Australia [5], 65% in the US [6], 64% in Mexico [7], 68.7% in Hong Kong [8], and 60% in Canada [9]. Primary dysmenorrhea refers to painful menstrual cramps in the lower abdomen without evident pelvic pathologic lesions. This condition occurs just before or at the onset of menstrual flow. Females with primary dysmenorrhea produce excessive amounts of prostaglandins and leukotrienes in the uterus, and these substances cause inflammation, myometrial hypercontractility, and vasoconstriction [10, 11]. The uterus then becomes ischemic and hyperalgesic, resulting in cramps and systemic symptoms such as nausea, vomiting, and headache. Dysmenorrhea can affect mental concentration during class, restrict social activities, reduce academic achievement [12], increase absenteeism [1, 7, 13], and reduce quality of life [4]. Previous studies have demonstrated that primary dysmenorrhea changes central sensitization to pain perception and alters brain metabolism, particularly in gray matter [14]. These findings indicate that the adolescent brain is sensitive and susceptible to menstrual pain [15].

Nonsteroidal anti-inflammatory drugs (NSAIDs) and oral contraceptives (OCs) are the most commonly used and effective pharmacologic treatments for pain relief from primary dysmenorrhea [16, 17]. In this study, 12–28% of adolescents sought medical care and 60.9–66.9% of adolescents self-medicated themselves with over-the-counter drugs [7, 18, 19]. Although NSAIDs provide temporary relief from menstrual pain, they may have side effects such as gastrointestinal upset, indigestion, headaches, and drowsiness [20]. Moreover, OC usage is significantly related to higher incidences of irregular uterine bleeding and nausea. Adverse events are more common during the early stage of use [21], and endometriosis is more frequent with long-term use [22]. In traditional Chinese medicine, the acupoint stimuli transmitted to the brain and specific organs in the rest of the body can modulate physiological reactions [23]. This causes the release of various neurotransmitters, which interrupt afferent signals in the central nervous system [24]. Auricular acupressure is a simple noninvasive method of acupoint stimulation. Auricular acupressure can be used to treat the symptoms associated with primary dysmenorrhea, inhibit excessive production of prostaglandins, reduce excitability of the cerebral cortex, and regulate endocrine hormone secretion [25]. Many studies have demonstrating the beneficial effects of auricular stimulation on menstrual pain and distress [26–30].

Most adolescents lack appropriate and sufficient information about menstruation and must be empowered to take charge and manage their own care. Adolescents with dysmenorrhea often receive information about dysmenorrhea from school [3], their mothers, siblings and friends, physicians and nurses [19], and others [8]. Of these sources, health care professionals are the most flexible and adaptable in their approach to providing patients with information [31]. Internet-based systems are flexible communication methods that deliver the latest, most up-to-date information [32], and their use for pain management has become popular [33]. Previous studies using Internet-based learning programs have reported reduced pain [34–36], facilitated knowledge acquisition [37], facilitated self-care education [38], and improved health status [39]. However, the effects of auricular acupressure combined with Internet-based programs on menstrual pain and self-care for dysmenorrheic adolescents may be more comprehensive. 

## 2. Purpose Statements

This study investigates the effects of auricular acupressure (AA) alone or when combined with interactive Internet-based (II) interventions on menstrual pain and self-care in adolescents with primary dysmenorrhea. Significant differences in scores on the short-form McGill pain questionnaire (SF-MPQ), visual analogue scale (VAS) for pain, menstrual distress questionnaire (MDQ), and adolescent dysmenorrhea self-care scale (ADSCS) were hypothesized between and within the groups.

## 3. Methods

### 3.1. Research Design and Participants

**Figure 1:**
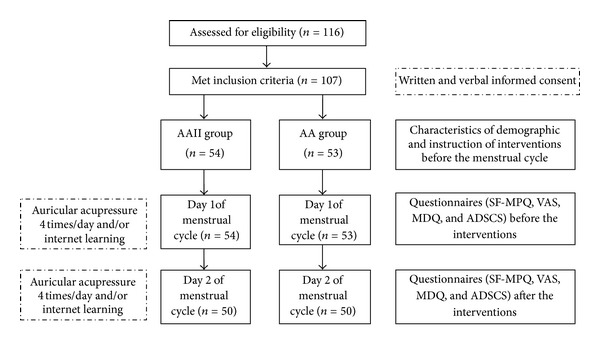
The Flow Chart of Research Design and Participants.

This study adopts a pretest/posttest control design involving 107 participants with primary dysmenorrhea from a senior high school. We divided participants into two groups. One group received auricular acupressure (AA) alone, whereas the other received AA combined with an interactive Internet-based (AAII) intervention.  Figure 1 shows a flowchart of the participants of this study. Inclusion criteria were (1) two or more incidents of menstrual pain experienced in the past six months; (2) VAS > 5; and (3) no swelling, infections, or ulcers in the bilateral ears. Exclusion criteria were (1) known diagnosis of pelvic inflammatory disease, endometriosis, or gynecological surgery, and (2) taking analgesic drugs or herbal medicine for dysmenorrhea. We used G Power version 3 to calculate the sample size and found that each group should contain 45 participants to achieve a statistical power of 0.80 with a statistical significance of 0.05. We calculated the effects of auricular acupressure on pain relief in adolescents with dysmenorrhea based on the study of Yeh et al. (in press). Pain relief (mean ± standard deviation) was 5.14 ± 2.32 in the experimental group and 3.64 ± 2.49 in the control group. We collected data before and after the interventions and compared the effects of interventions between and within the groups.

### 3.2. Intervention

Six auricular acupoints were used for relieving dysmenorrhea: *shenmen*, *kidney*, *liver*, *internal genitals*, *central rim*, and *endocrine*. The effects of stimulation depended on the following specific auricular acupoints: *internal genitals* and *endocrine* were for harmonizing and improving endocrine and uterine function [40], *kidney *and* liver* were for normalizing qi and blood and restoring organ function [41], *shenmen* was for alleviating pain and for sedation [29], and *central rim* was for dredging the meridian and normalizing circulation [42]. We adopted a seed-embedding method with cowherb seeds to stimulate the auricular acupoint. Two experts licensed in traditional Chinese medicine confirmed the accuracy and precision of seed positioning and pressing techniques. We placed adhesive plasters containing seeds on the auricular acupoint at the start of menstrual bleeding and removed after pain relief 48 hours later. All participants were instructed to press each acupoint for at least one minute, four times per day until experiencing pain relief.

We created an interactive website to promote the health of those with dysmenorrhea. This website provided clients with nursing care instruction and counseling and served as an interactive communication medium for increasing knowledge about dysmenorrhea and self-care practices related to dysmenorrhea. Two experts in the discipline of obstetrics and gynecology examined the content validity of the program and evaluated the correlation between objectives and content. These experts graded each of the 20 items on a four-point scale for item-objective congruence and relevance. The overall content validity index was 0.95. We divided the contents of dysmenorrhea into nine units as follows.

*Unit 1: Hot News*. To disseminate information and build consensus, this unit provided a preview of activities and reported the prevalence of dysmenorrhea, legal rights in the workforce relating to dysmenorrhea according to gender equality law, and the health-care concerns of adolescents. *Unit 2: Red Magic Book. *This included an online survey and menstrual diary. Participants were asked to fill out relevant questionnaires before and after self-care learning, and record the necessary information in their menstrual cycle diaries. This data was charted automatically to create a menstrual cycle chart. *Unit 3: Understanding of Dysmenorrhea. *We used computer-animated videos to describe the menstrual cycle and hormone fluctuation during ovulation and presented the definitions and differences between primary and secondary dysmenorrhea in table format. We also presented the physical and psychological symptoms of dysmenorrhea in interactive format and discussed the effects of dysmenorrhea on daily life. *Unit 4*: *Caring. *Menstrual care included menstrual care based on the viewpoint of Chinese medicine, self-care approaches such as hot pack, daily menstrual care, and menstrual hygiene. *Unit 5*: *Auricular Acupressure*. We used pictures with word descriptions to introduce acupoint techniques and the theory, rationale, efficacy, advantages, and precautions associated with these techniques. The photographs explained and illustrated the six auricular acupoint procedures for pain relief. *Unit 6: Professional Counseling. *Professional health-care providers responded to posted questions through this webpage or email. We prioritized questions and answers from participants to present their main concerns and issues. *Unit 7: Diet and Food Properties. *This part of the website presented content relating to daily diet requirements to preserve health, food properties based on the rationale of Chinese medicine, and general information on food properties and herbal cuisine dietetics. *Unit 8: Chat Room. *Using this part of the website, participants could post messages regarding dysmenorrhea or the menstrual cycle to interact with each other and provide support. *Unit 9: ext-linked Websites*. We also provided hyperlinks to representative and authoritative websites on dysmenorrhea to extend the learning experience.

### 3.3. Measures

We used the short-form McGill pain questionnaire (SF-MPQ) developed by Melzack [43] to assess the quality and intensity of pain. The SF-MPQ includes an 11-item sensory subscale and four-item affective subscale rated from 0 to 3 (none, mild, moderate, and severe). The Cronbach's alpha for this measure was 0.84 in this study. We also assessed pain intensity on a 0–100 mm visual analog scale (VAS), with the left end labeled as no pain and the right end labeled as unbearable pain. All participants rated their present pain intensity. The test-retest reliability of the VAS for pain was 0.97 [44].

We used the modified 16-item menstrual distress questionnaire (MDQ) developed by Wang [45] to assess the severity of physiological symptoms (pain, water retention, and autonomic reactions) during the premenstrual and menstrual periods. Participants graded each item on a four-point scale from one (no symptoms) to four (severe symptoms). Cronbach's alpha for this measure was 0.83 in this study. Additionally, we used the adolescent dysmenorrheic self-care scale (ADSCS) with seven subscales (including search for knowledge, expression of emotions, seeking assistance, control over external factors, resource usage, self-control status, and Internet information) to evaluate self-care behaviors [46]. Participants graded each item on a six-point Likert scale. We added six items to the 40 original items to confirm the interactive content. Cronbach's alpha for this measure was 0.90 in this study.

### 3.4. Data Analysis

We analyzed data using IBM SPSS 20.0, and used descriptive statistics to describe demographic characteristics. We also used the chi-square test, paired *t*-test and one-way ANCOVA to analyze the effects of interventions on these characteristics between or within groups. We considered a *P* value of less than 0.05 to be statistically significant.

## 4. Results

The sample in this study initially consisted of 107 participants. Of these, seven participants withdrew from the study for personal reasons (*n* = 5) or because they had difficulty using computers (*n* = 2). Finally, each group consisted of 50 participants, with an attrition rate of 6.5%. The average ages of the AAII and AA groups, respectively, were 16.94 ± 1.02 and 17.94 ± 0.84 years at the beginning of the study and 11.96 ± 1.21 and 12.18 ± 1.56 years at menarche. The mean length of the menstrual cycle was 28.82 ± 3.74 and 29.70 ± 4.02 days.  Table 1 presents a summary of the demographic characteristics of groups at baseline and shows no significant between-group differences, except in age (*P* < 0.001) and menses duration (*P* = 0.008).

Table 2 shows the improvement in pain management after the AAII and AA interventions. The between-group difference was not significant in pre-to-posttest change in SF-MPQ (*P* = 0.81), VAS (*P* = 0.75), and MDQ (*P* = 0.28) scores. The within-group difference in pre-to-posttest change in SF-MPQ, pain VAS, and MDQ scores was significant for both the AAII (*P* < 0.001) and AA (*P* < 0.001) groups.  Table 3 presents a summary of the results of ADSC scale for dysmenorrhea. We found a significant difference in pre-to-posttest change in the total scores between the groups (*P* < .001) and in each group (AAII: *P* < .001; AA: *P* = 0.04). The between-group differences in pre-to-posttest change in scores for all subscales (*P* < 0.05) and the within-group difference in pre-to-posttest change for all subscales in the AAII group (*P* < 0.001) were significant. However, these differences were significant only in the searching for knowledge (*P* < 0.001) and self-control status (*P* = 0.02) subscales in the AA group.

## 5. Discussion

Most of the participants in this study started with menstrual pain within two years after their menarche and experienced dysmenorrhea within the first two days of menstruation. Other studies have reported similar findings [3, 4, 7, 8, 20, 47, 48]. In this study, the average age at menarche was approximately 12 years, which is slightly lower than that in Turkey (13.38; [4]), Iran (13.3 years; [49]), Korea (13 years; [50]), Nigeria (12.7 years; [13]), Japan (12.5 years; [21]), and Hong Kong, South Africa, and Mexico (12.3 years; [8, 18, 47]). However, early menarche is defined as menarche beginning before age 11-12 [51–53].

The results of this study show that auricular acupressure alone or combined with interactive Internet instruction can reduce menstrual pain and distress. This observation is consistent with previous studies [26, 28, 30]. Auricular acupressure combined with interactive Internet intervention was more effective than auricular acupressure alone in improving dysmenorrhea self-care. Auricular acupressure alone, compared to analgesics, achieved greater improvement in menstrual pain and associated syndromes [29, 54–56]. Auricular stimulation of local pressure receptors results in nerve impulse transmission, and pain decreases or disappears when the intensity of the stimulus exceeds a threshold. Based on this mechanism, auricular acupressure can relieve menstrual pain and distress in adolescents with primary dysmenorrhea.

The interactive Internet-based intervention proposed in this study provided knowledge and information about dysmenorrhea self-care, auricular acupressure techniques, professional counseling, and peer support. Our results are consistent with previous findings showing the efficacy of Internet-based interventions in reducing pain [35, 36, 57, 58] but are not in agreement with the findings of Trautmann and Kröner-Herwig [59]. A systematic review shows that Internet-based programs appear to relieve pain [33]. Autonomic reactions refer to pain, stress, and anxiety [60]. A program of interactive online learning may reduce autonomic reactions by increasing knowledge about self-care techniques, thereby reducing pain and anxiety [34, 57] and stress [57, 61]. Future research should measure autonomic reaction indicators, such as heart rate variability, to clarify the mechanisms involved in menstrual distress and autonomic nervous system activity.

This study also shows that auricular acupressure combined with interactive Internet instruction is better than auricular acupuncture alone in improving self-care for primary dysmenorrhea. Other studies have also shown that Internet- or computer-based interventions enhance knowledge and technical abilities or promote self-care ability in patients dealing with idiopathic carpal tunnel syndrome [62], adolescents preparing for out-patient tonsillectomy procedures [37], and patients attempting to self-manage chronic low back pain [63]. A systematic review of relevant literature shows that Internet-based interventions used to disseminate information on treatment are an effective complementary tool for changing lifestyle habits, diminishing symptom severity, and improving decision-making skills [64]. Thus, Internet-based interventions could be integrated into programs that enhance the knowledge and self-care behaviors of adolescents with primary dysmenorrhea. Overall, the proposed treatment improves self-care behaviors in both the AAII and AA groups, and especially the AAII group. Interactive Internet education programs not only provide knowledge of self-care activities, but also lead to increased self-efficacy [39] and confidence in the use of self-care techniques [38]. Therefore, Internet interventions can increase the effectiveness of auricular acupressure.

### 5.1. Limitations

This study has some limitations. First, nonrandomized clinical research involves inherent limitations. Second, this study does not include a control or placebo group; therefore, the results of a placebo effect are unknown. Third, the sample was only taken from one senior high school, which may limit the generalizability of the results. Fourth, this study only demonstrates the short-term effects of the proposed interventions; the long-term effects remain unknown. Fifth, we collected menstrual pain and distress data through self-reported questionnaires and did not measure physiological indicators. Randomized controlled, longitudinal studies that measure physiological indicators in various geographic locations are recommended. In addition, future research should address the current limitation of accessing interactive Internet-based interventions in the AA group.

## 6. Conclusion

This study contributes information regarding the effectiveness of employing auricular acupressure combined with interactive Internet-based instruction and auricular acupressure alone in adolescents with primary dysmenorrhea. Our results show that auricular acupressure improves menstrual distress and self-care behavior. The interactive Internet-based intervention in this study, which is flexible and available to adolescents seeking information to manage health-related issues, generated even more efficient self-care behaviors. However, the long-term effects of auricular acupressure combined with interactive Internet-based intervention and auricular acupuncture alone in adolescents with primary dysmenorrhea remain unclear. Future studies should integrate Internet-based interventions with other interventions to improve the self-care of menstrual pain and distress in adolescents. In addition, objective measures of the autonomic nervous system activity (physiological indicators) are needed to enhance the value and reliability of this type of intervention.

## Figures and Tables

**Table 1:** Comparisons of demographic characteristics between the groups.

Variables	AAII (*n* = 50)	AA (*n* = 50)	*t *
	Mean (SD)	Mean (SD)	
Age (years)	16.94 (1.02)	17.94 (0.84)	0.75**
Age at menarche (years)	11.96 (1.21)	12.18 (1.56)	2.46
Menstrual cycle (days)	28.82 (3.74)	29.70 (4.02)	0.29
Menses duration (days)	5.86 (1.16)	5.24 (1.12)	0.002*

	*n* (%)	*n* (%)	*χ* ^2^

Menstrual regularity			0.00
Yes	30 (60.0)	30 (60.0)	
No	20 (40.0)	20 (40.0)	
Initial onset of menstrual pain			0.35
Menarche	13 (26.0)	11 (22.0)	
<1 year after menarche	15 (30.0)	15 (30.0)	
1-2 years after menarche	10 (20.0)	12 (24.0)	
Others	12 (24.0)	12 (24.0)	
Time of dysmenorrhea			0.25
Day before menses	11 (22.0)	9 (18.0)	
First 2 days in menses	39 (78.0)	41 (82.0)	

AAII: auricular acupressure combined with interactive internet; AA: auricular acupressure.

*P < 0.05.

**P < 0.001.

**Table 2:** Outcomes on auricular acupressure combined with interactive internet or alone.

	AAII (*n* = 50)			AA (*n* = 50)			ANCOVA
Variables	Pre-test	Post-test	Improvement	Pre-test	Post-test	Improvement	
	Mean (SD)	Mean (SD)	Mean (SD)	Mean (SD)	Mean (SD)	Mean (SD)	*F*
SF-MPQ	16.65 (8.88)	4.58 (3.73)	11.98 (8.46)^a∗∗^	18.80 (9.26)	5.36 (6.76)	13.44 (8.62)^a∗∗^	0.06
VAS	6.56 (1.36)	1.97 (1.87)	4.59 (1.93)^a∗∗^	7.17 (1.46)	2.03 (2.02)	5.14 (2.32)^a∗∗^	0.11
MDQ	29.30 (7.12)	21.88 (7.06)	7.42 (9.28)^a∗∗^	31.88 (7.60)	21.16 (4.67)	10.72 (6.85)^a∗∗^	1.18

SF-MPQ: short-form McGill pain questionnaire; VAS: visual analog scale; MDQ: menstrual distress questionnaire.

^a^Within-group differences.

**P < 0.001.

**Table 3:** Results of adolescent dysmenorrhea self-care scale.

	AAII (*n* = 50)			AA (*n* = 50)			ANCOVA
Variables	Pre-test	Post-test	Improvement	Pre-test	Post-test	Improvement	
	Mean (SD)	Mean (SD)	Mean (SD)	Mean (SD)	Mean (SD)	Mean (SD)	*F*
Searching for knowledge	11.54 (4.63)	18.54 (2.94)	7.00 (5.13)^a∗∗^	13.54 (4.68)	15.46 (4.89)	1.92 (3.06)^a∗∗^	32.67**
Expression of emotions	24.66 (6.53)	28.50 (4.93)	3.84 (5.09)^a∗∗^	29.58 (5.57)	29.02 (6.37)	−0.56 (4.59)^a^	7.49*
Seeking assistance	16.68 (4.21)	19.08 (3.34)	2.40 (3.30)^a∗∗^	18.88 (4.40)	18.66 (4.15)	−0.22 (3.31)^a^	8.77*
Control over external factors	20.52 (5.65)	26.06 (5.71)	5.54 (5.90)^a∗∗^	23.68 (7.48)	24.62 (7.03)	0.94 (3.96)^a^	14.52**
Resource utilization	47.64 (11.29)	62.12 (16.11)	14.48 (15.63)^a∗∗^	57.20 (10.69)	58.00 (10.87)	0.80 (6.52)^a^	18.02**
Self-control being	14.86 (5.07)	19.32 (5.01)	4.46 (4.76)^a∗∗^	16.12 (4.88)	17.50 (3.86)	1.38 (4.18)^a∗^	10.55*
Internet information	14.34 (5.65)	27.16 (5.47)	12.82 (7.16)^a∗∗^	19.64 (7.44)	21.14 (5.50)	1.50 (6.20)^a^	50.67**

Total score	150.24 (30.94)	200.78 (32.25)	50.54 (30.16)^a∗∗^	178.64 (28.60)	184.40 (28.27)	5.76 (18.80)^a∗^	46.92**

^a^Within-group differences

*P < 0.05.

**P < 0.001.
